# Recent Advances in Click Chemistry Applied to Dendrimer Synthesis

**DOI:** 10.3390/molecules20059263

**Published:** 2015-05-20

**Authors:** Mathieu Arseneault, Caroline Wafer, Jean-François Morin

**Affiliations:** Département de Chimie, Université Laval, 1045 avenue de la Médecine, Pavillon Alexandre-Vachon, QC G1V 0A6, Canada; E-Mails: mathieu.arseneault.1@ulaval.ca (M.A.); caroline.wafer.1@ulaval.ca (C.W.)

**Keywords:** dendrimer, dendron, click, Hüisgen cycloaddition, thiol-ene, thiol-yne, Diels-Alder

## Abstract

Dendrimers are monodisperse polymers grown in a fractal manner from a central point. They are poised to become the cornerstone of nanoscale devices in several fields, ranging from biomedicine to light-harvesting. Technical difficulties in obtaining these molecules has slowed their transfer from academia to industry. In 2001, the arrival of the “click chemistry” concept gave the field a major boost. The flagship reaction, a modified Hüisgen cycloaddition, allowed researchers greater freedom in designing and building dendrimers. In the last five years, advances in click chemistry saw a wider use of other click reactions and a notable increase in the complexity of the reported structures. This review covers key developments in the click chemistry field applied to dendrimer synthesis from 2010 to 2015. Even though this is an expert review, basic notions and references have been included to help newcomers to the field.

## 1. Introduction

The click chemistry paradigm, published in 2001 [1], was quickly applied to the synthesis of new dendritic architectures [2]. Among the criteria that define click chemistry—high reaction enthalpy, high chemoselectivity, atom economy and environmental safety—the first two combined to unlock new structures that would have been otherwise unattainable. In recent years, great strides have been accomplished in the field of dendrimer synthesis and we have witnessed an increase in the complexity of the reported assemblies. Fine-tuning of reaction conditions proper to the field also resulted in noteworthy achievements. Herein we review the advances published between 2010 and early 2015 featuring click chemistry applied to dendrimer synthesis or functionalization. Our starting point is the 2010 review from the Kakkar group that had a similar focus [3]. The first aim of the present review is to offer an update of the field regarding new dendritic constructs, whether they pertain to new architectures or to a new level of complexity in design. A line had to be traced and we chose to limit ourselves to well-defined dendrimers and dendrons. Hyperbranched polymers, pseudo-dendrimers and star polymers are excluded because the lack of monodispersity in these architectures frees them from certain design constraints found in dendrimer synthesis. The synthesis of these macromolecules presents other challenges that do not overlap entirely with synthesis of dendrimers. For readers interested by that topic, there are excellent review articles [4,5,6]. The second goal of this review is to catalog the reaction conditions used during the years covered. Many of these have not been developed within our time scope, but they are still in use and therefore relevant to report and discuss. Special attention is also paid to the dendrimers that make specific usage of 1,2,3-triazoles. The shortness of this article forced us to make editorial choices. Nevertheless, over three hundred articles were surveyed and analyzed to write this focused review. Before discussing the novelty in a field that is now more than a decade old, basic notions must be covered, however briefly.

## 2. Click Reactions Applied to Dendrimers

### 2.1. Dendrimers

**Figure 1:**
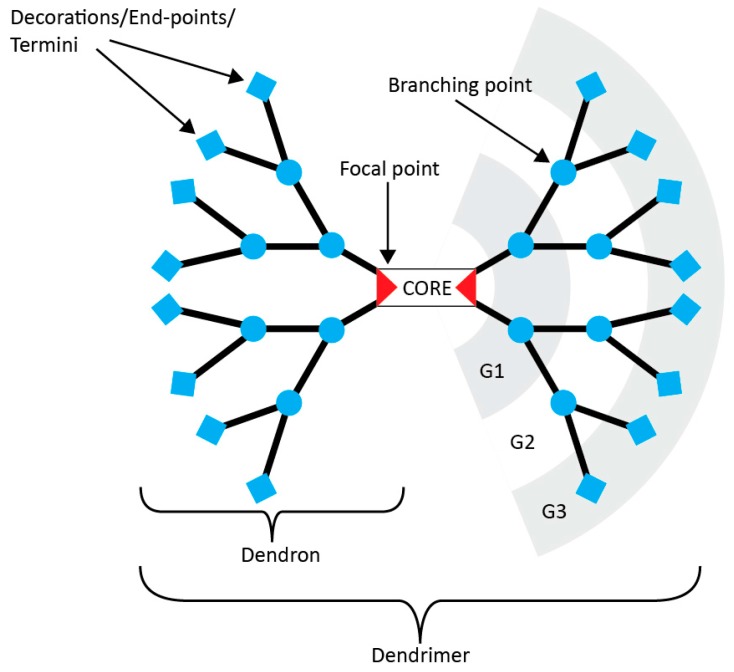
Anatomy of a dendrimer. This example is an AB_2_ type, where each branch is made of a monomer A that splits into two B monomers. “G” stands for generation.

A dendrimer is a regularly branched macromolecule that splits in a controlled manner to form what resembles a tree, hence the use of the Greek root for the word tree “*dendr*”. Counting Tomalia’s initial paper on poly(amido amine) (PAMAM) where the word was coined as the starting point [7], dendrimers are now thirty years old. Numerous books [8,9,10] and about 22,000 articles have since been published on the topic. Their physico-chemical properties diverge so greatly from classic polymers that they are often regarded as a separate class of molecules. These differences stem almost entirely from three key features: their monodispersity, their multivalency and their globular shape. Each of these advantages has been harnessed with great imagination. The applications range from biomedical devices [11,12,13] to light/energy harvesting [14,15] and catalysis [16]. Yet, very few products utilizing dendrimers and only one drug have reached the market [17]. The main reason beyond this is the synthesis of dendritic scaffolds themselves. The need for highly efficient reactions that are orthogonal to each other and the difficulties to purify an increasingly ramified macromolecule have hindered transfer from academia to industry. Figure 1 presents the anatomy and the vocabulary of dendrimer chemistry.

### 2.2. The Click Chemistry Paradigm Applied to Dendrimers

Click chemistry was elaborated first and foremost around several high yielding reactions. While 95% conversion is sometime considered “complete” in organic chemistry, it is not sufficient to build a dendrimer, especially higher generation ones. Completion at each step is crucial to maintain the purity and consequently the monodispersity of a dendrimer. Added to this stringency, the iterative two-step process in either convergent or divergent synthesis requires sturdy orthogonality. Few reactions feature both yields over 99% and good orthogonality to each other [18]. This is why click chemistry has been key to many new dendritic architectures. The atom economy and the environmental friendliness that are also criteria for a reaction to be deemed “click” are of a much lesser importance in the dendrimer community, at least from our survey. Application of click chemistry to material chemistry beyond dendrimers has been skilfully reviewed by several teams [19,20,21].

#### 2.2.1. Copper-Assisted Azide-Alkyne Cycloaddition (CuAAC)

**Scheme 1:**
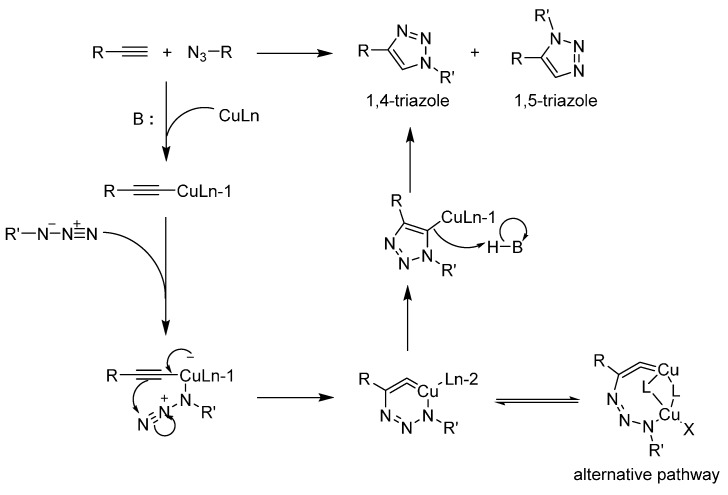
The Hüisgen [2 + 3] cycloaddition and the monomolecular version of the mechanism when it is catalyzed by copper. The alternative dinuclear transition state presented here is now widely accepted and explored by several groups [23,24,25,26].

Sharpless *et al*., reported a modification of the Hüisgen [2 + 3] cycloaddition between an alkyne and an azide catalyzed by copper under mild conditions (Scheme 1) [22]. It was published quickly after the article describing the click concept and the reaction became so closely associated with it that the term “click” is now often used to describe this specific reaction. It accommodates both hydrophilic and hydrophobic substrates, a precious characteristic for amphiphilic macromolecules. It can also operate in a wide range of pH values (5–12) and proceeds at room temperature. The catalyst pair is quite benign and cheap compared to a vast majority of organometallic compounds. In light of all these advantages, it is not surprising that these conditions are still the most used by a wide margin. This reaction is now so common that it is often viewed as a non-issue. Yet, on many occasions, it needs to be optimized.

Despite the sturdiness and versatility described above, classic CuAAC conditions can sometimes fail, especially in dendrimer synthesis and decoration. The most common shortcoming is that the 5% copper sulfate/10% sodium ascorbate catalyst is insufficient to complete the reaction. Three main reasons can explain this problem. First, the catalyst load has to be adjusted as a function of the number of reacting sites on a given dendrimer, leading to catalyst loads that can become almost stoichiometric in regard of a single site [27,28,29,30,31]. The percent mol ratio is specified to be per coupling site in some cases and per mol of substrate in others, or simply unspecified, leaving a significant margin to be re-optimized by others. Second, metal complexation by the dendrimer substrate can become an issue depending on the nature of the branches. Catalyst has to be added until the substrate is filled. This leads to problematic purifications as discussed below. Third, deactivation of the Cu(I) catalyst to Cu(0) or Cu(II) is more likely to happen with a higher number of reacting sites and longer reaction times [26]. Beside adding more copper, the usual two-fold excess of sodium ascorbate may be increased to five-fold [30,32] to ensure the copper remains in the proper oxidation state. Interestingly, the exhaustive work of the Lee group shows a clear example where yields either decrease as generations go up under a given set of conditions [29], or that longer reaction times and heating have to be applied to maintain yields [33,34]. It should be noted that systematic investigation of reaction conditions is a rare thing in dendrimer synthesis mostly because of the time and costs involved in preparing substrates that can hardly relate to one another. The speed of the reaction is akin to a second order reaction in regard to the catalyst concentration [35]. Unfortunately, some dendrimers are only soluble at low concentrations, owing to their high molar mass and rigidity. This constitutes another argument in favor of a higher catalytic load. Oxidation by ambient air can shorten copper catalysts’ lifespan over longer reaction times [26]. Oxidised copper can then lead to acetylene homo-coupling (Glaser-Hay reactions), destroying two molecules of the alkyne substrate at each turnover [36]. Carrying out the reaction under an inert atmosphere usually solves the issue.

The hydrophilicity of the solvents can sometime be unsuitable for highly hydrophobic dendrimers. Commonly used hydrophobic conditions featuring CuBr as the copper source is listed in Table 1 alongside all the noteworthy conditions we found. It should be noted that this condensed table only covers the last five years and readers should also consider Meldal’s detailed 2008 review for more conditions [37].

**Table 1:** CuAAC conditions noted in dendrimer synthesis from 2010 to 2015.

Catalyst	Base/Acid/Reductant/Ligand	Solvent	Temp.	Type of Architecture	Typical Reference
CuSO_4_	NaAsc, THPTA, TBTA, Ph-COOH, K_2_CO_3_, NaHCO_3_	H_2_O, THF, DMF, *t*-BuOH ^a^, EtOH ^a^, MeOH ^a^	rt, 50 °C, 85 °C ^b^, MW	PAMAM, Fréchet-type, Percec-type, polyesters, PEG branches, polypeptides, others	[30,31,38,39,40]
CuOAc	NaAsc, THPTA, TBTA	DMF, MeOH	rt, 100 °C	Polypeptides, others	[39,41]
CuI	Et_3_N, DIPEA	DMF, THF, DCM	rt	PAMAM, polyesters, polypeptides, others	[40,42,43,44]
CuBr	PMEDTA	DMF, THF	rt, 40 °C	PEG-branches, others	[38,45,46]
CuF_2_	-	MeOH, H_2_O	40 °C	Polyesters	[40]
Cu(MeCN)_4_PF_6_	DIPEA	MeOH, DCM	rt	Peptides	[47]
Metallic Cu	Et_3_NH_4_Ac	MeOH	MW (7h), 70 °C	Phosphocarbohydrates	[48,49]

^a^ It has been reported that alcohols can slow the reaction, despite being frequently used [50]; ^b^ These conditions were reported to be possible due to a protection of end groups on a PAMAM [27].

The groups that first described CuAAC are still investigating its mechanism. The latest advance favors a dinuclear mechanism featuring two copper ions and two sets of substrates [51,52,53]. It is also now believed that several closely related mechanisms could be operating alongside one another. This mechanistic flexibility partially explains the efficient conversion observed in CuAAC. Astruc provided an interesting exploration of this and proposed several alternatives in his seminal 2011 article [54]. It is most likely that steric hindrance shifts the mechanism towards a mononuclear one as the dendrimer generation increases. Another key aspect of the mechanism is the need for a base to deprotonate the alkyne and help the copper to bond at the end of it. While CuAAC has been shown to proceed at pHs as low as 5, it is often carried out at pH 8 to 10. Bases usually include carbonate salts and secondary or tertiary amines. In 2010, Shao *et al*., screened several weak organic acids added to CuAAC [55]. The argument for this modification is that a source of proton helps complete the catalytic cycle by removing the copper from the triazole. This strategy was applied to the synthesis of at least two dendrimers [27,56]. Given that there are few cases featuring this addition, it is too early to predict which dendritic architectures will benefit from it.

A key improvement of CuAAC over the original Hüisgen cycloaddition [57] is the possibility to perform the reaction at room temperature. Originally, the activation barrier was so steep that high temperatures had to be maintained for the reaction to proceed. It also yielded a mixture of 1,4 and 1,5-triazoles (Scheme 1) while CuAAC only produces the 1,4-regioisomer under the proper conditions. For dendrimer synthesis, the 1,5-triazole could be much more difficult due to sterical hindrance and potentially change the behavior and accessibility of the decorating moieties. This is most likely why we could not find dendrimers assembled through the ruthenium version of CuAAC (RuAAC) since it selectively produces the 1,5-regioisomer [58]. We noted a few examples where CuAAC carried out at 50 °C still leads to the 1,4-triazole [59,60]. In such cases, the steric hindrance that slows the reaction and warrants an increase in temperature also greatly favors the proper regioisomer. In other cases, people have used microwave heating to reach high temperatures over a short period of time [30,32,61,62,63,64].

As stated earlier, copper removal can be a critical issue. Dendrimers complex copper [65,66] and other metals so efficiently that they are often studied as a support to grow metallic nanoparticles [67]. Copper contamination is a concern mainly because of its *in vivo* toxicity [68] and its ability to quench fluorescence [69]. Several purification methods to remove copper from dendrimers have been reported and can be found in Table 2. While these procedures are sometime able to remove the quasi-totality of copper ions, their chelating ability can be surpassed by the dendrimer itself. Predicting the chelating power of a given architecture is a difficult task. Many factors come into play, but the presence of tertiary nitrogen atoms near the branching points that can act as pincers like those found in PAMAM is a good indicator. Triazoles can also act as chelating points [25].

**Table 2:** Methods used to remove copper from CuAAC in dendrimer synthesis from 2010 to 2015.

Method	Comment	Typical Reference
NH_4_Cl sat. washes	This is a pH 5 solution that can be basified by adding NH_4_OH. We found that several washes were needed.	[44]
EDTA washes	Because of EDTA four pKa values, we observed a great variability of pH and efficiency, depending on the commercial version used.	[70]
KCN washes	Given the high toxicity of HCN, this solution needs to be kept basic until safely discarded.	[71]
Dialysis	This method is the longest and suits hydrophilic dendrimers that are unfit for aqueous/organic washes. Dilute EDTA can be added to the external solution.	[72,73]

**Figure 2:**
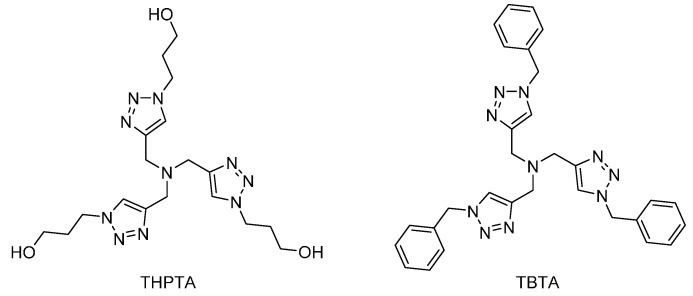
Tris(3-hydroxypropyltriazolylmethyl)amine (THPTA) (left) and tris-(benzyltriazolylmethyl)amine (TBTA) (right). Similar ones have been developed and studied as CuAAC ligands [25].

An alternative solution adopted by researchers, especially in the biomaterial community, is to use a strongly chelated source of copper. Doing so not only prevents the dendritic substrate from capturing copper ions, but also keeps the metal in the desired oxidation state as well [25]. Steric hindrance may also favors the 1,4 isomer. The most used ones for dendrimer synthesis are shown in Figure 2. They can be removed by conventional methods such as column chromatography or aqueous washes. Two points can be derived from their structures: they are dendritic in nature and they rely on nitrogen complexation and not oxygen. This is in good agreement with a conclusion from Shao *et al*., who observed that acetate ligands are likely to be detrimental to CuAAC completion [54]. In 2011, Astruc further explored CuAAC through the design of dendritic catalysts [53]. Their work resulted in another small dendritic catalyst, dubbed Cu-tren, showing great promise both in activity and in ease of preparation. Larger dendritic [16,53,69] or polymeric [74,75] catalysts are often reported, but have yet to be used in a routine manner.

**Figure 3:**
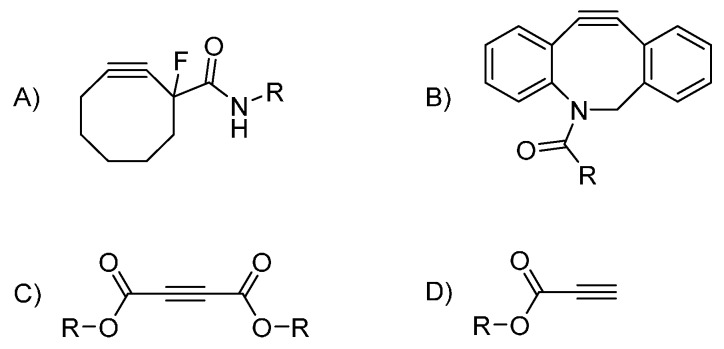
Copper-free synthons used in dendrimer synthesis (**A**) fluorinated cyclooctyne (**B**) a dibenzoazocyclooctyne variant (**C**) acetylenediesters (**D**) propiolic esters.

A well-established trend in azide-alkyne click chemistry is to forego metal catalysis altogether. Bertozzi developed a strategy relying on cycle tension release of a cyclooctyne to activate the alkyne (Figure 3) [76,77]. Strain promoted azide-alkyne cycloaddition (SPAAC) is now routinely used in dendrimer chemistry, mostly for final decoration purposes [78,79,80]. Its orthogonality with CuAAC has proven to be useful in creating multifunctional substrates [81,82] or Janus dendrimers [68]. As a testament to the utility of SPAAC, Bertozzi’s synthons are now commercially available. Nevertheless, their bulkiness and hydrophobicity [83] added to the fact that it can hardly branch out could explain why we did not find any dendritic architecture built around it. The Baker group used a cyclooctyne bearing a fluorine atom in the vicinal position of the alkyne. This stems from the computational study carried by Goddard III [84] and experimental work by Bertozzi [77]. It showed that having electron- withdrawing groups (EWGs) conjugated with the alkyne greatly activated SPAAC. Sharpless himself noted early on that EWGs were efficient activators [22]. Application of this strategy to dendrimer synthesis is a much more recent development though. Acetylenedicarboxylic esters have been used to first decorate a highly congested low generation dendrimer by Fessner [85] and then for the branching points by Brook [86] and ourselves [87]. While very successful, these endeavors are still isolated cases. Acetylenedicarboxylic esters are notably delicate and much work remains to be done to transform the idea into a more flexible strategy. As a side note, we found a few interesting optimizations regarding the installation of azides onto dendritic scaffolds. The temperarture if the usual S_N_2 reaction carried out in DMF at *ca.* 80 °C can be lowered to 40 °C [88] or even room temperature in some cases [89]. Other azides can also be used [90] under milder conditions. In the same line of thought, Hamilton used a succinimide-based azide at room temperature [91].

It should be noted that the Hüisgen cycloaddition, with or without copper, features some dangers. It is exothermic and can reach hazardous temperatures with highly activated substrates. In our hands, the EWG strategy self-heated to an estimated 120 °C in seconds and charred itself. Added to this, activated small moieties (such as acetylenecarboxylates) that react spontaneously can be highly toxic until transformed into the triazole. Moreover, synthesis and use of labile azides need to be planned carefully to prevent toxic and explosive decompositions. Usual laboratory precautions are sufficient to tackle said risks.

#### 2.2.2. The Thiol-ene and Thiol-yne Click Reactions

**Scheme 2:**
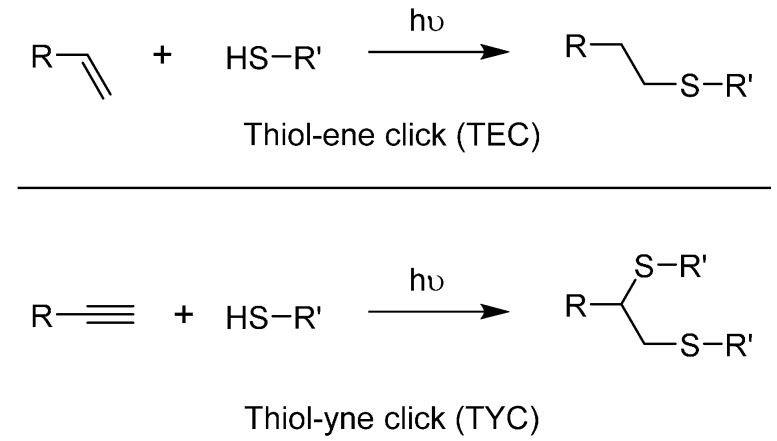
General schemes for the thiol-ene click reaction **(**Top) and for the thiol-yne click reaction (Bottom).

The origin of the thiol-ene click reaction (TEC) is more diffuse than CuAAC as Hoogenboom and Lowe pointed out [92,93], but it has now evolved into a well-defined set of conditions. In its current form, TEC consists of the reaction between a terminal alkene and a thiol as shown in Scheme 2. It can either proceed through a free-radical mechanism or a nucleophilic one [94]. The former is the most popular since it can be photocatalyzed. Classic conditions are mild and orthogonal to a large spectrum of reactions, including the Michael Addition and CuAAC. All these advantages combined qualify this reaction to be seen as a “click” one. TEC was covered in details in several recent reviews [93,95,96,97,98,99,100] and we focus only on recent application to dendrimer synthesis. The wavelengths used to initiate the reaction vary from 350 to 365 nm. For dendrimer synthesis, having fewer reactants leads to easier purifications, which can be crucial. That said, our survey showed that in most cases, a radical initiator was needed to bring the reaction to completion. Notably, 2,2′-dimethoxy-2-phenylacetophenone in substoichiometric quantities was used for this purpose in recent works [101,102,103]. It can be carried out in a large selection of solvents (THF, MeOH, H_2_O, DMF, DCM), usually under an inert atmosphere to avoid side reactions involving molecular oxygen. Precipitation in ether and chromatography columns are the most used purification methods we noted. Recently, Bowman *et al*., screened the reactivity of various vinyl and thiol units in a dendritic setting [104]. Despite being newer and less popular than CuAAC, TEC now has its place in the toolbox of material chemists. Its tolerance toward other reactions has led to various pairs of reactions for the iterative synthesis of dendrimers in recent years. Key examples are discussed in a later section.

The parent thiol-yne click reaction (TYC) functions through the same mechanism [92,105]. The difference is that once a thiol fragment has linked to the alkyne, a second reaction can occur on the resulting alkene. TYC is much more difficult to control in terms of regioselectivity, even more so if one aims to prevent the addition of the second thiol. This challenge is almost irrelevant in dendrimer chemistry though, as people have been using it mainly in a branching strategy [92,106,107]. By using the same conditions than TEC, two thiol moieties react in a predictable manner onto a terminal alkyne (Scheme 2). TYC branching points are represented as only one diastereoisomer, but researchers acknowledge the 1:1 ratio between the *trans* and *cis* isomers [103]. The effect or absence thereof of this mixture on the final functionality is sometime discussed by researchers, but without a noticeable trend yet given how recent this strategy is. While both TEC and TYC can be carried out on substituted moieties, we noted only terminal examples. TYC offers a noteworthy advantage of being orthogonal with CuAAC while sharing its substrates, namely the alkynes. Examples of how this was successfully applied to glycodendrimers are available in Section 3. Biocompatibility of TYC and TEC was partially covered in a review regarding the role of sulfur in glycodendrimers [108].

#### 2.2.3. The Diels-Alder (DA) Reaction

**Scheme 3:**
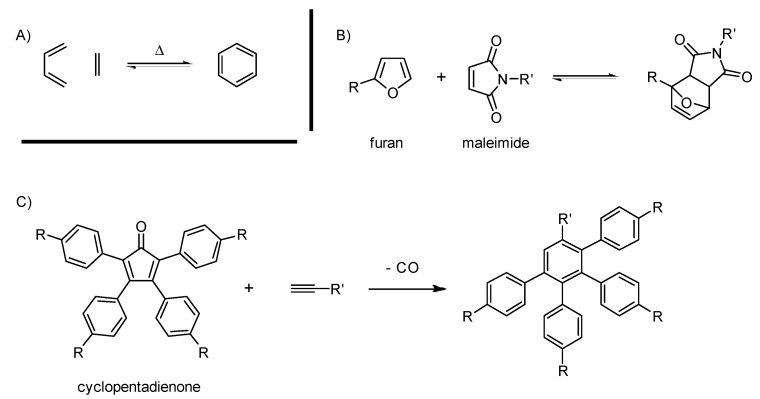
General scheme for the Diels-Alder (DA) reaction (**A**), an example of rDA with furan and maleimide (**B**) and the cyclopentadienone popularized by the Mülllen group (**C**).

The reaction between a diene and a dienophile (Scheme 3) viewed through the “click” lens has been covered recently [20,109]. The main point is that DA meets the convenient advantages offered by click reactions through atom economy, high conversion, simple conditions and good chemo and regioselectivity. Another feature of the reaction is the possibility to choose whether it is reversible or not by carefully selecting the precursors. Müllen popularized the use of cyclopentadienone as the diene to build carbon-rich dendrimers [110]. During this specific case, a carbon monoxide molecule is lost, preventing any retro Diels-Alder (rDA). According to our survey, the conditions to carry out this reaction have not evolved noticeably within the last five years, except for the use of microwave irradiation to reach 300 °C [111]. With this optimization, Bunz *et al*., were able to reach new levels of steric hindrance in a dendrimer. For them, DA was more reliable than organometallic coupling. Carbon-rich materials regularly feature melting points reaching 350 °C, so the high temperatures needed are not an issue. Noteworthy structures published since 2010 using this strategy are detailed in Section 3.

DA is also used to synthesize biocompatible macromolecules [109,112]. Here, high temperatures can become problematic (loss of secondary and tertiary conformation, thermolabile bonds, *etc*.). The use of highly activated substrates alleviates this issue. For this purpose, maleimide, furan [113,114,115,116,117,118] and anthracene [115,117,118,119] remain the most popular ones. The compatibility of CuAAC and DA was used in several cases. Meanwhile, examples of orthogonality between DA and TEC are far rarer, but do exist. Interestingly, Bowman *et al*. used TEC to block the retro aspect of DA between furan and maleimide [120]. The retro character of these two synthons is well-known and is at the center of some recent self-immolatives dendrimers as discussed later on.

#### 2.2.4. Upcoming Click Reactions for Dendrimers

Despite the progress of the three click reactions described above, there will always be room for new synthetic tools. Ever-growing orthogonality “unlocks” more complex materials that can be used as a lab-on-a-molecule [121], performing several reactions and detections in parallel. Beyond the need for multifunctionality, synthetic chemists need to be able to vary the nature of the linking groups at will.

Majoral and Caminade have been exploring phosphorus chemistry applied to dendrimer synthesis for several years now [122]. One of their most recent development modifies the Staudinger reaction to give it “click” attributes [123]. A Janus [123] and an “onion peel” [124] dendrimer were built with this new reaction as proof-of-concept. Other reactions that features click characteristics are being investigated for materials synthesis, such as the *trans*-cyclooctene/tetrazine [125] pair and Sharpless’ new SuFEx reaction [126]. Both could potentially be used in dendrimer synthesis. Other potential “click reactions” have been covered by Hoogenboom [127].

## 3. Structural Advances

### 3.1. Rise of Complexity

The extended family of macromolecules bearing dendrons have been reviewed abundantly over the years [9]. Thus, we chose to focus on select aspects of novelty in recently reported structures. Nevertheless, seemingly classical designs sometime require unusual strategy and need to be discussed. The last five years have witnessed a notable increase in the complexity of published dendritic structures. Complexity often implies great difficulties in material synthesis and this is where click chemistry truly shines. Deconstructing the paradigm, one can see how features of click reactions are left behind in order to keep only the ones that unlock target structures.

#### 3.1.1. Difficult Decorations and other High Molar Mass Dendrimers

Obtaining a high molar mass dendrimer may not seem like a noteworthy feat at first glance, but in order to maintain the polydispersity index under 1.1, there are certain milestones that can halt progress. Novel solutions need to be developed, either in reaction conditions or characterisation techniques. A good example is the new megadalton dendrimers published by the Müllen group [128,129]. They used the same cyclopentadienone strategy described above, but changed the shape of the branches and managed to reach 100 kDa. Extreme steric hindrance at the periphery of a dendrimer is often pointed as the main obstacle toward very high generation dendrimers or bulky decoration. Efficient conversion, especially in the divergent method, then becomes essential. CuAAC is often used to decorate dendrimers. Its efficiency allows the use of voluminous moieties that would otherwise be very difficult or simply impossible to put on a dendrimer. Zong, in collaboration with Baker went a step further by doing a two-stages post-functionalization of a PAMAM [81]. Other large decorations that were enabled by click chemistry include peptides [32,130,131,132,133,134], carbohydrates [104,135,136,137,138,139,140,141,142,143,144], DNA [140,145,146], sRNA [147] and chelated gadolinium [131,134]. In that area, Cloninger *et al*., proposed a new way to envision surface functionalization of a dendrimer using CuAAC [148]. Departing from the conventional idea of uniformly capping end groups, they proposed to actively seek a clustering of a given decorating moieties. This was done by building a smaller dendron bearing several copies of the first moieties. This clustering created larger “free zones” at the periphery, allowing for a secondary bulky decoration. Interestingly, Wagner *et al*., decorated a dendron with oligoamines that proved incompatible with subsequent CuAAC, presumably because of copper trapping [149]. The solution was simply to switch the synthesis order. Fessner *et al*., screened several activated alkyne for CuAAC in their 2010 article and they managed to functionalize a POSS core with adamantane units [85]. The article of Bunz discussed above can also be seen as a type of difficult decoration that was enabled by click chemistry [111]. In a publication by the Tang group, propiolic acid was used with a source of copper to activate the reaction both through the catalytic and the EWG strategies at the same time to add a bulky decoration on a congested G4 dendrimer [150].

#### 3.1.2. Janus and Controlled 3-Face Dendrimers

**Figure 4:**
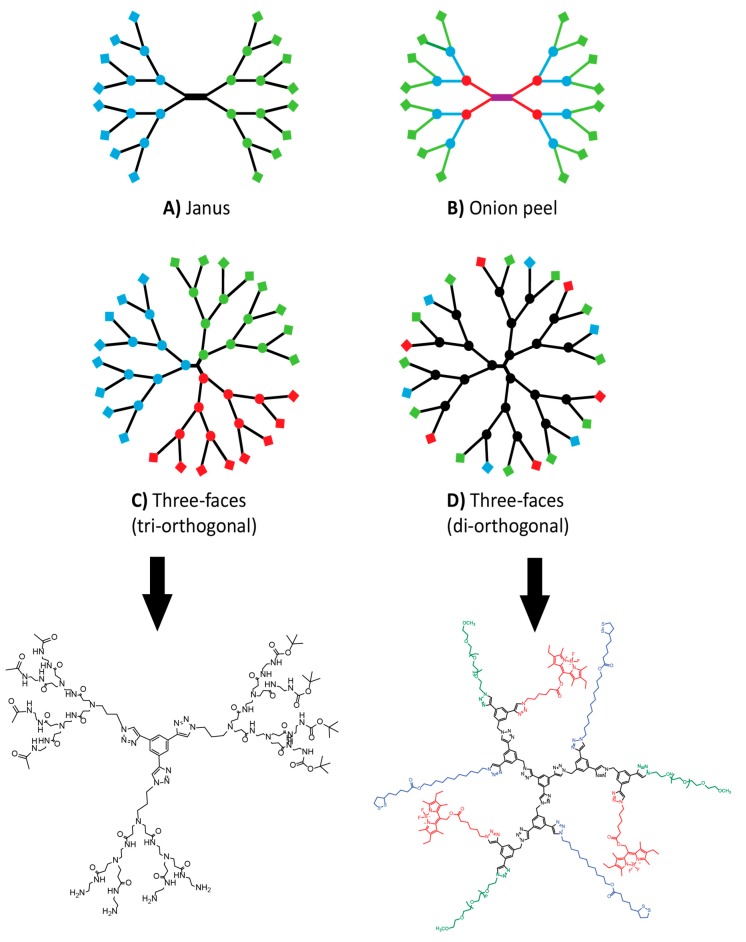
Complex dendritic architectures evolution from the pre-existing Janus (**A**) to the “onion peel” (**B**) and the three-faces strategies that rely on tri-orthogonality (**C**) or di-orthogonality (**D**).The structures of C and D are shown below to highlight how CuAAC was used only at the core for C and within the branches for D.

The work of Cloninger cited above is an evolution of the common statistical decoration of a given dendrimer with multiple moieties. Multifunctional dendrimers have been studied extensively and will certainly play a key role in upcoming medical devices [11,151,152]. That said, this approach has some caveats. Complete conversion of end groups is very difficult to achieve when sequentially adding moieties [12,81,153,154]. This leaves unreacted sites that need to be capped so that they do not react afterward and results in handful of active moieties (5/64 for example) statistically distributed on the surface of the dendrimer. In his 2012 review, Grinstaff made a point that the interest in dendrimers over other polymers is their monodispersity [11]. Therefore, statistically decorated dendrimers partially undermine this advantage. As depicted in Figure 4, several alternative architectures have been published where each dendron of a given dendrimer is fully decorated with a different species. Janus or bow-tie dendrimers were published before the advent of click chemistry [155], but it allowed greater flexibility around the concept. Extensive work by the Lee group and others produced a variety of structures. Among these, dendrimers of the same nature, but with different generations [59,156], same generation with different architectures [34,157], both [71,123,158] or different decorations [73] were reported over the last five years. The chemoselectivity and orthogonality of click reactions are instrumental in achieving these structures. In each case, monodispersity was maintained. A unique attempt at employing dendrimers to make molecular barcodes was published by the Hawker group [159]. CuAAC allowed them to fully decorate their dendrimer with different ratios of (*R*) and (*S*)-groups.

Going back to the lab-on-a-molecule concept, there is a need for dendrimers bearing three or more well-defined faces. Orthogonality issues long barred the way to such structure, but from 2010 to 2012, several approaches were published on this concept, some featuring click reactions. Fukase *et al*., were the first ones to offer a monodisperse dendrimer bearing three different decorations [160] and Kakkar *et al*., expanded on the concept in 2011 [161]. They used an A(BC) approach where each branch in a branching point can be addressed independently, thus placing three types of decorating species while using a two-fold orthogonality. A few months later, we used the variety of available alkyne protecting groups as a source of three-fold orthogonality. It resulted in a controlled three-face PAMAM dendrimer [27]. As a side note, Weck [162] and Rudick [163] published syntheses of three-face dendrimers using non-click methods during the same period.

#### 3.1.3. The “Core-Shell-Surface” and the “Onion Peel” Strategies

In 2011, the Müllen group described a layered dendrimer in which the core, the generations and the decorations are closely related, yet structurally different [164]. They dubbed this strategy “core-shell-surface” and used it to give the dendrimer complex optical behavior. Others in that field followed this idea [165,166]. In the biomedical area, CuAAC was used in the synthesis of a very large (47 kDa) polypeptide dendrimer by Gu [60]. This dendrimer also has layered generations, each having a different structure. In 2014, the Roy group added to the concept by developing the “onion peel” strategy [102,103]. Here, each generation is not only structurally different from the others, but also relies on a different reaction. CuAAC, TEC/TYC and esterification were used in a single dendrimer. The potential advantages to this approach all revolve around the fine tuning of physico-chemical properties. Said properties will be the result of a balancing act between the layers. The idea was undoubtedly enabled by the orthogonality of click reactions and has already been picked up very recently by the Majoral group [124].

#### 3.1.4. Advances in Dendronized Polymer Synthesis

Dendronized polymers are notoriously difficult to synthesize [167,168,169]. The wedge shape of the grafted dendrons prevents them from being close to each other along the main polymer chain. Many reactive sites are thus left untouched. Dynamic folding of the main chain in solution further affects the availability of said sites. This yields polymers with a very large polydispersity index. Alternatively, trying to polymerize dendrons through their focal point leads to a very low degree of polymerization. The third option is to grow the dendrons directly unto the polymer, but then growing steric hindrance can lower the purity of neighboring dendrons. Click chemistry alleviates some of these issues and teams now often opt for the first strategy, “clicking” premade dendrons unto the main chain. Most of these newly reported polymers used standard CuAAC [117,170,171,172,173,174], but others used DA [115,117,118,119,175] or TEC [176]. Here, the orthogonality and the efficient conversion of click reactions are both needed. Sanyal *et al*., harnessed the orthogonality of CuAAC and DA to assemble a dual-dendron dendronized polymer [117]. McElhanon also used CuAAC to add dendrons onto thermosensitive polymers built with click DA [114,116]. In a step-up of complexity, Sanyal and Rotello decorated a polymer with flavin units through CuAAC, which were further functionalized with dendrons through non-covalent bonds [175].

#### 3.1.5. Vesicles and Micelles

The scientific community regularly produces complex macromolecules and supramolecular assemblies featuring dendrimers. Between 2010 and 2014, the Percec group, along with several collaborators, reported the creation of dendrimersomes [177,178]. Mimicking the behavior of phospholipids in cell membranes, amphiphilic dendrimers were synthesized. Their architecture and generations were varied, some even used TEC. It was observed that by varying the solvent, a plethora of self-assembled nanostructures ranging from disks to dendrimersomes could be obtained. The Roy group joined this multi-team endeavor and made amphiphilic glycodendrimers with CuAAC [179]. The resulting vesicles exhibit novel properties and trends, different from their dendritic constituents. As the authors explained, these assemblies could become the cornerstone of new biotechnologies featuring complex cell-like devices. Other groups have used click chemistry to assemble micelles from dendrimers. Thayumanavan used a complex asymmetric dendron to first create the micelle through self-assembly [180]. Then, UV light was used to crosslink the interior, trapping the guest molecule. Meanwhile, the Toth team used a long chain dendrimer decorated with peptides that self-assembled into a micelle that expressed the peptides at its surface [48].

#### 3.1.6. Degradable Dendrimers

Self-immolative dendrimers occupy a growing niche in the scientific literature [181,182]. Born from the degradation studies of common dendrimers such as PAMAM and PPI, the field now actively seek to create dendrons made of weak covalent bonds. Programmable host delivery, tunable biodegradability and timed bio-release (like a spaceship shedding its rockets in stages) are example of applications. This is a stark departure from a common goal in material chemistry; to find sturdy links that can withstand the most conditions possible. Since CuAAC and TEC/TYC produce stable bonds, it may explain why click chemistry is very rarely used [183]. Nevertheless, the groups of Kakkar [113] and Hoogenboom [119] both employed rDA as the sensitive linkage. You used TEC to add a crosslinking ability to his immolating dendrimers [184].

#### 3.1.7. Other Complex Assemblies

**Scheme 4:**
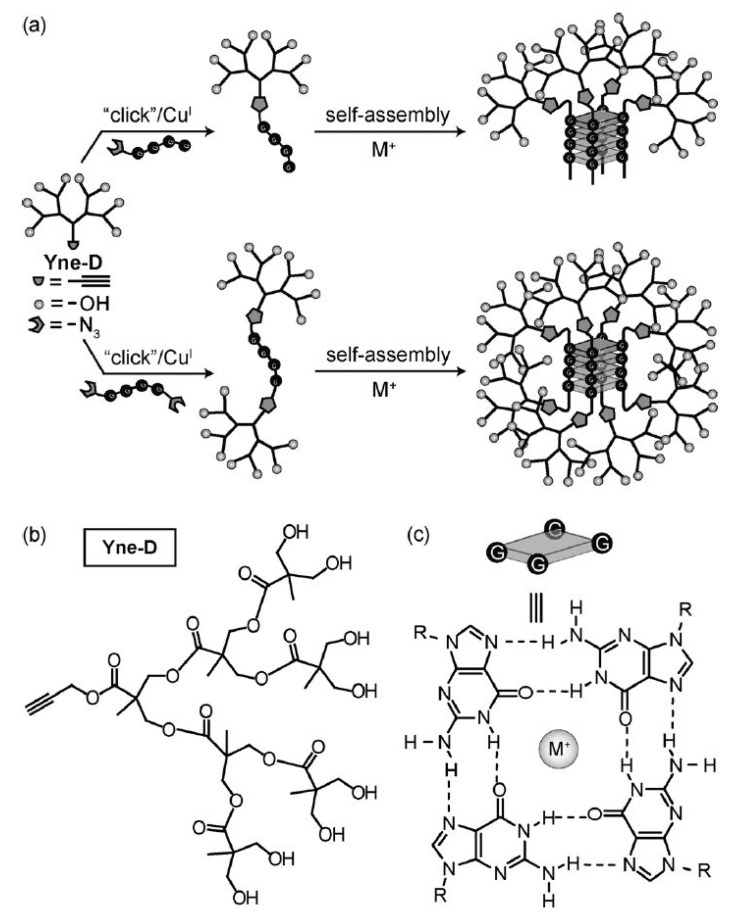
Dendrons with an alkyne focal point are clicked on a nucleotide strand which ends bear azides. The global strategy where the Yne-D dendron is “clicked” on either a mono or di functionalized nucleotide strand is shown in (**a**). the structure of the Yne-D dendron is shown in (**b**) while (**c**) shows the detail of the nucleotide self-assembly around the metal ion. The resulting dendrimer self-assemble around a metal ion to form a cubic core with a total of four or eight dendrons. Reproduced with permission from Hamilton *et al.* [91].

Entire dendrimers utilized as the building blocks of a larger system include the work of Holl [153], who used SPAAC to link together PAMAM dendrimers [82]. In a similar fashion, dendrimers can act as crosslink points to synthesize dense network polymers. CuAAC [185], TEC [104] and TYC [106] have all been used for this purpose in recent years. Malkoch *et al*., also combined CuAAC and TEC to fabricate a hydrogel centered on dendrimers [186]. Going in a different direction, Hamilton *et al*., reported the synthesis of oligoguanosines capped with clicked dendrons (Scheme 4) [91] These macromolecules self-assembled in a cube bearing a dendron at each corner. The structures were thermosensitive and combinatorial chemistry was performed. Nucleotide delivery and sensing could be achieved with this approach.

When coating a metallic surface or nanoparticles (NPs), most teams tether dendrons to the surface by the focal point. They do so to orient the end-groups outside, toward the solution. Inversely, researchers also use the termini as a multi-foot anchor [187,188]. In all cases, standard CuAAC was used without issue within these very complex systems. Two original articles concerning the synthesis of rotaxanes relied on CuAAC to install dendrons as end blockers [33,189]. Interestingly, the Lee group used a cucurbituril macrocycle that was not only part of the final rotaxane, but catalyzed CuAAC at the same time [33]. Finally, an original scaffold in which dendrimers line up on a DNA strand through strand association was reported by Gothelf *et al*. [146]. Half of the G4 PAMAMs were decorated with alkynes and the other half with azides. Once they were each associated at their respective places on a single strand bearing all the codes, CuAAC was performed to solder the dendrimers to each other. Linear and circular shapes were made using this method to demonstrate its ability to template large dendrimers.

### 3.2. New Dendritic Architectures Featuring Click Chemistry

Each year, several new dendritic architectures are published. Researchers either seek to adapt existing architectures to their specific needs or to overcome the synthetic problems that are common to all dendrimers. Nowadays, such problems are mainly purity at high generation, convenience and efficiency. The last two may seem frivolous, but they are important issues that still need to be overcome before new dendrimer-based devices can reach mass market applications [190,191]. This section focuses on the new architectures that employ a click reaction in at least one of the iterative step. Furthermore, click chemistry opened the possibility of modifying with a certain ease pre-existing architectures without major changes. This is why we choose to focus on novel strategies rather than specific branches.

**Scheme 5:**
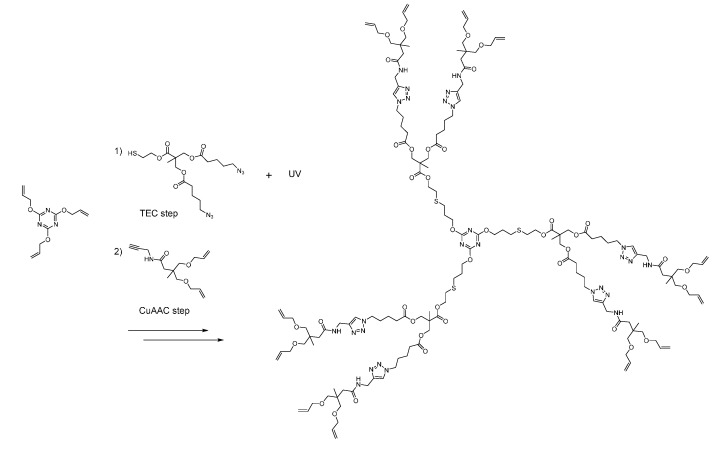
The global strategy of Malkoch *et al*. Through careful synthesis of starting synthons, they were able to use CuAAC and TEC without any transformation. This allowed for a very convenient and fast divergent synthesis [190].

Over the last five years, there has been a net increase in the number of dendrimers relying on either TEC or TYC. The two reactions were coupled to several other reactions such as Michael Addition [192], CuAAC [103,193], epoxy opening [194], esterification [192,195,196] and silane chemistry [197,198]. In 2009 and 2010, silane chemistry was used with either Grignard [199] or NaBH_4_ [200], demonstrating the sturdiness of the thioether. Soon after, Hawker and Malkoch published a combination of esterification, TEC and CuAAC in a AB_2_/CD_2_ strategy [190]. They were able to quickly reach the 6th generation in a single day, much faster than other synthesis (Scheme 5).

**Scheme 6:**
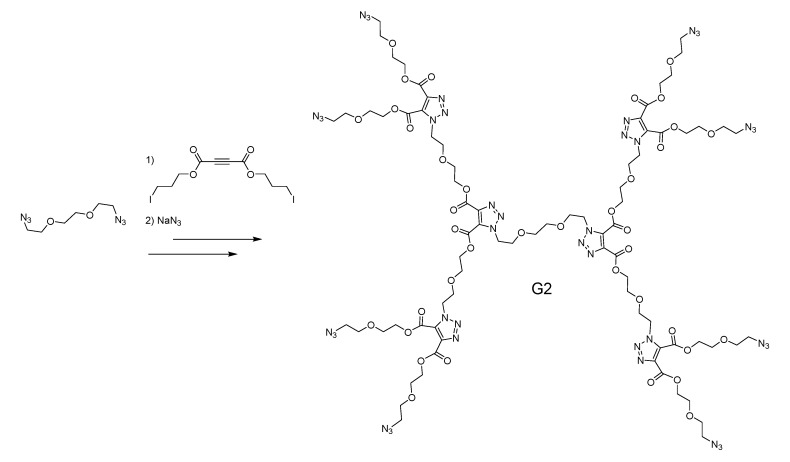
A metal-free dendrimer assembled through the EWG-alkyne-azide strategy [87]. If the substrates are oils at room temperature, no solvent is needed and the reaction can be self-heated.

The polydispersity index rose from 1.02 to 1.21 between G4 to G5, signaling a critical point in steric hindrance at the periphery. Notably, they specifically chose not to perform chromatography at any step. Since then, Dondoni used it to make a glycodendrimer [193]. Afterward, Hawker published architectures, this time using TYC and epoxy opening [107,194]. An interesting feature of this dendrimer are the hydroxyl groups that are left free at each generation. As a proof-of-concept, they attached fluorophores to them. Since 2010, there have been some interesting cases of novel architectures involving CuAAC beside those mentioned in the paragraph above. First, Harriman *et al*., built a unique dendrimer made of porphyrines as the branching points in an AB_3_ fashion [90]. The core and each generation had its own metal core, leading to a complex electronic behavior. Second is the nitrone branching strategy presented by Junkers *et al.* [201] Here, a AB/CD_2_ approach involving a seldom-used radical reaction combined with CuAAC afforded dendrimers up to G3. This is the only dendritic architecture we encountered that features N-O bonds. Third, Fokin [202] and Kakkar [203] both published similar carbon-rich scaffolds, using Sonogashira coupling on aromatic units to build their monomers. Finally, the Li group has published a series of dendrons and dendrimers built for non-linear optics purposes. Variations included internal decorations such as sulfate and nitro [204], but they could also swap the cores [205] and the terminal decorations [206], all thanks to the versatility of CuAAC. As discussed earlier, the groups of Brook and Morin published new methods of building dendrimers through highly activated alkynes (Scheme 6) [86,87]. These copper-free methods were developed with biomedical aims by purposefully avoiding metals from beginning to end. Early cytotoxicity results validate this approach [87]. Aspects of convenience and speed were also highlighted in these works.

From time to time, the triazole group is sought after for its electronic properties. Astruc and others published a large body of work regarding dendrimers in which the triazole is conjugated to the decorating ferrocenyl [207,208,209,210,211,212,213,214,215,216] and cobalticinium [217]. This conjugation was found to have a positive influence on the redox ability of these dendrimers. Moreover, it was observed that the triazole can play an important role in catalysis and in the formation of Pd NPs. Another unique entry by Astruc *et al*., used the triazole as a covalent nucleation agent to grow gold nanoparticles [218]. The triazole can exhibit antifungal properties and is found in many synthetic drugs as well. These roles go beyond the scope of this review and have been detailed elsewhere [219,220].

## 4. Conclusions

In this focus review, we reported recent advances in click chemistry applied to dendrimer synthesis. Special attention was given to current and novel reaction conditions in CuAAC, TECTYC and DA. Recent applications of these three reactions were then reviewed. A few overarching trends could be identified. First, while CuAAC remains the most used of all click reactions, it is now losing ground to the other ones and to its copper-free variants. SPAAC is now commonly used to decorate dendrimers and a few novel architectures relying on highly activated alkynes were recently published. Copper removal continues to be a concern in dendrimer synthesis, but there is now a wide array of methods available. The second major trend is that between Kakkar’s 2010 review and now, TEC went from “novel” to “well-established”. The combination of all the new dendritic architectures using TEC or TYC constitute a useful body of knowledge. It will be interesting to see which, if any, will come out as the most popular. Asking this question, though, might quickly become irrelevant as we see a shift from the type of branches to rather a given set of iterative reaction that are not tied to a specific monomer. Going even further, the “onion peel” strategy showcased how click chemistry enables a high degree of customization of dendritic architectures. The third trend our survey highlighted is how click chemistry is now a philosophy that extend beyond its own reaction. Case in point, the Michael Addition, a reaction that predates click, is now described as being click. Within the scope of dendrimer synthesis, we noted a deconstruction of the paradigm with the catalytic and the environmental friendliness set aside when needed. Reactions are sometime seen as having click-like characteristics [127]. As a final note, our survey picked many articles about hyperbranched polymers arguing that dendrimer synthesis is too difficult. This next-door-neighbor look offers a good indication that the field still needs to strive for greater ease. Click chemistry is instrumental in tackling these issues. As reported here, great progress has been accomplished in a short time.
